# MiR-487a Promotes TGF-β1-induced EMT, the Migration and Invasion of Breast Cancer Cells by Directly Targeting MAGI2

**DOI:** 10.7150/ijbs.67062

**Published:** 2021-09-21

**Authors:** Mengtao Ma, Miao He, Qian Jiang, Yuanyuan Yan, Shu Guan, Jing Zhang, Zhaojin Yu, Qiuchen Chen, Mingli Sun, Weifan Yao, Haishan Zhao, Feng Jin, Minjie Wei

**Affiliations:** 1 Department of Pharmacology, School of Pharmacy, China Medical University, Shenyang, Liaoning Province, China; 2 Department of Surgical Oncology, The First Affiliated Hospital of China Medical University, Shenyang, Liaoning Province, China

In our paper 1, Figure 3 should be corrected as follows.

## Figures and Tables

**Figure 3:**
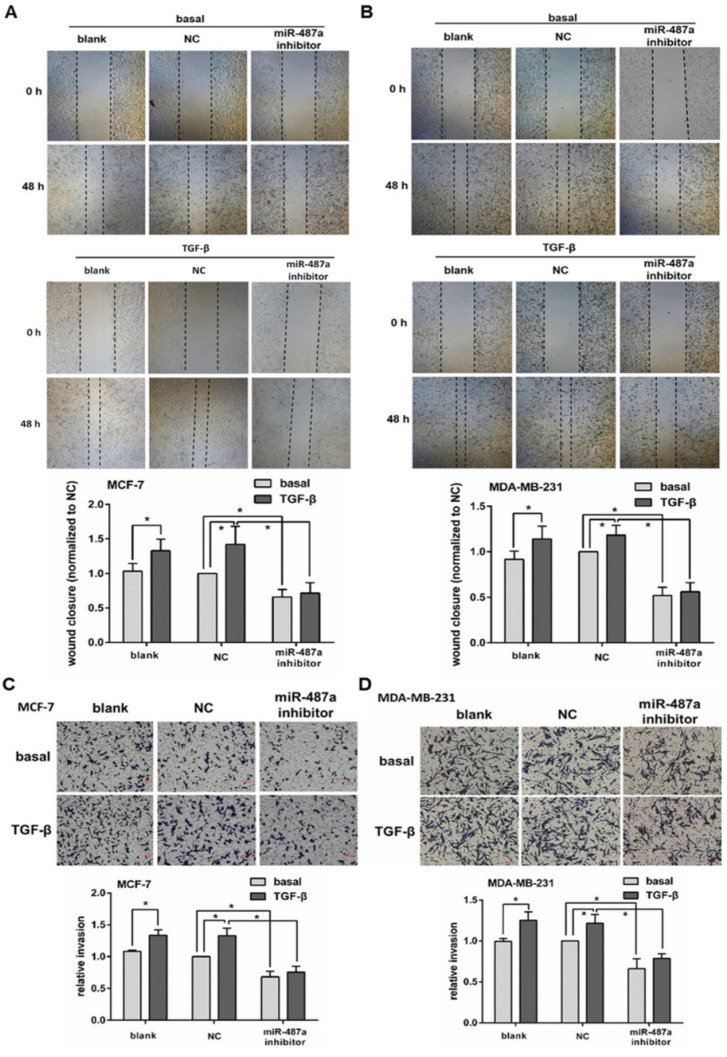
** The down-regulation of miR-487a inhibits the migration and invasion induced by TGF-β1 in breast cancer cells. The migration abilities were measured by wound healing analysis** in MCF-7 cells (A) and MDA-MB-231 cells (B) transfected with miR-487a inhibitor or NC, and treated with or without TGF-β1. The wound closure in the cells transfected with NC and treated without TGF-β1 was set 1. The invasion abilities were measured by transwell invasion assay in MCF-7 cells (C) and MDA-MB-231 cells (D) transfected with miR-487a inhibitor or NC, and treated with or without TGF-β1. The invasion ability of the cells transfected with NC and treated without TGF-β1 was set 1. *P<0.05.
